# Medical Society of the County of Erie

**Published:** 1896-02

**Authors:** 


### Buffalo Medical Journal.

A Monthly Review of Medicine and Surgery.

*EDITORS:*

THOMAS LOTHROP, M. D. - - WM. WARREN POTTER, M. D.

All communications, whether of a literary or business nature, should be addressed to the managing editor: 284 Franklin Street, Buffalo, N. Y.

Vol. XXXV. FEBRUARY, 1896. No. 7.

### MEDICAL SOCIETY OF THE COUNTY OF ERIE.

### A DIAMOND ANNIVERSARY.

When a medical society has reached an age that enables it to celebrate its seventy-fifth anniversary, the occasion is unusual enough to merit more than passing comment. Though a full account of the proceedings held by the Medical Society of the County of Erie at its annual meeting, January 14, 1896, when it also celebrated its seventy-fifth anniversary, is published in its appropriate place in this issue of the Journal, we feel that befitting remark thereon should be made here.

In the first place, the arrangements for the commemorative part of the meeting were placed in the hands of a committee, consisting of Drs. Lucien Howe, chairman, Charles G. Stockton, P. W. Van Peyma, J. W. Putnam and William H. Gail, that performed its duties in a most satisfactory manner. It was a most appropriate beginning for Dr. Franklin C. Gram, the secretary, to lead off with a historical sketch. His work was gracefully and felicitously done. Dr. Gram succeeded in grouping a vast array of historical material condensed into narrow compass, and he interspersed it with delicious bits of humor that prompted his audience to punctuate the reading with frequent bursts of applause.

When the venerable Dr. John Hauenstein took the rostrum to read his paper on The first uses of chloroform and ether in Buffalo, he was accorded a reception that was as flattering as it was deserved. During the reading of his paper, too, there was frequent applause, especially when mention was made of the men who were prominent in those days.

Following this came Dr. C. C. Wyckoff, with an account of The establishment and early days of the Medical Department of the University of Buffalo. Here again was a subject of absorbing interest, and Dr. Wyckoff was accorded the just recognition of frequent applause during the presentation of his subject.

Finally, Dr. John Cronyn appeared upon the scene with an account of The establishment of the Medical Department of Niagara University, in which he felicitously told the story of the founding of this college. He, too, was greeted cordially and applauded handsomely.

In response to a circular, requesting photographs and biographical sketches of members, the secretary has received many responses, but is desirous that all who have not already done so shall promptly send to him their pictures and biographies. These will be appropriately filed in albums and volumes specially prepared for the purpose, and will form an interesting historical collection.

A number of members were invited to prepare sketches of medical events in Erie county with which they have been specially identified, and these are also to be carefully preserved for information and future reference. When these papers are all completed and filed with the secretary they will furnish a most interesting, instructive and valuable chapter in the medical history of Western New York.

It was voted at the meeting to publish the papers contributed in the BUFFALO MEDICAL JOURNAL and to print 500 extra copies in pamphlet form for distribution to the members, historical societies, libraries and medical societies in the state of New York.

This society, from its earliest history, has always taken a prominent part in medical affairs. Within its folds have emanated many questions of scientific importance, and it has conceived and carried forward to completion some of the most important legislation on the statute books, of which not the least significant is the present medical practice act, whereby the state exercises control over the right to practise by issuing a license, after due examination, to those who hold legal diplomas. It was fitting that a society with such traditions should commemorate its seventy-fifth anniversary in such a graceful and unostentatious manner.

The society seized upon this auspicious occasion to put in motion the machinery for the establishment of a professional home for its members and which shall include all medical bodies within the borders of the county. A committee was appointed, consisting of Dr. Ernest Wende, chairman ; Dr. Herman Mynter, president of the Academy of Medicine ; Dr. J. G. Thompson, president of the Medical Society of the County of Erie ; Dr. Henry Lapp, of Clarence, and Dr. Walter D. Greene, of Buffalo, which is empowered to consider the feasibility of building a medical club house and the methods of providing ways and means for the same, and is directed to report at the semi-annual meeting in June, 1896. We commend to the medical profession of the county of Erie this most important subject, and bespeak for this representative committee a liberal support in its laborious enterprise.

Finally, the chair was authorised to appoint a committee of five to investigate and report at a subsequent meeting upon the propriety of separating the governing power of the Almshouse and the County Hospital. The president, Dr. J. G. Thompson, subsequently appointed the following-named physicians to serve on this committee : Dr. Edward Storck, chairman, Drs. John J. Walsh, Wm. H. Gail, Henry Lapp and Henry R. Hopkins.

The 14th day of January, 1896, was a red letter day in the annals of the medical profession of Erie county, and the officers and committees of this ancient and honorable society, as well as all others who contributed to make it so interesting, are entitled to the praise and congratulations of the whole community.

